# Supplementary material for: The genome sequence of the Kentish Glory, Endromis versicolora (Linnaeus, 1758) (Lepidoptera: Endromidae)
Source: Wellcome Open Res. 2026 Aug 27;11:559. [Version 1] doi: 10.12688/wellcomeopenres.27493.1 (PMC13616053; doi:10.12688/wellcomeopenres.27493.1)
Supplement: Peer review history

# Reviewer report: Reviewer response for version 1

**DOI:** 10.21956/wellcomeopenres.30261.r168065

**Published:** 2026-09-26

**Authors:** Alena Suchackova Bartonova

**Affiliations:** 1 Biology Centre of the Czech Academy of Sciences, České Budějovice, Czech Republic

This is a report on the genome assembly of *Endromis versicolora* produced by the Darwin Tree of Life project. It provides background information on the species’ biology. PacBio and Hi-C sequencing were performed, and two haplotypes were assembled, with genome sizes of 391.9 and 391.4 Mb and BUSCO completeness scores of 98.9% and 98.8%, respectively. I have no requests for changes.

Are sufficient details of methods and materials provided to allow replication by others?

Yes

Is the rationale for creating the dataset(s) clearly described?

Yes

Are the datasets clearly presented in a useable and accessible format?

Yes

Are the protocols appropriate and is the work technically sound?

Yes

Reviewer Expertise:

entomology, molecular ecology, biogeography

I confirm that I have read this submission and believe that I have an appropriate level of expertise to confirm that it is of an acceptable scientific standard.

# Reviewer report: Reviewer response for version 1

**DOI:** 10.21956/wellcomeopenres.30261.r167214

**Published:** 2026-09-24

**Authors:** Vlad Eugen Dinca

**Affiliations:** 1 Muzeul National de Istorie Naturala Grigore Antipa, Bucharest, Bucharest, Romania

In this data note, the authors have successfully generated a chromosome-level genome assembly of a male *Endromis versicolora* (Lepidoptera: Endromidae). The mitochondrial genome has been assembled as well.

 Although I am not an expert in several of the methodological approaches used, as far as I can tell, the manuscript is technically sound and uses methodologies and pipelines that are fairly well-established through use by the Sanger Institute.

 The genome appears to be of high quality and scaffolded into 26 chromosomal pseudomolecules, including the Z sex chromosome.

 Reference genomes such as this one represent a valuable resource for the scientific community and each new addition provides new opportunities for research.

 A few additional comments are included below.

 Background, first line: It could also be said that this species is the only European representative of the family Endromidae.

 Background: Perhaps it would be good to also include the scientific names of the host plants. It may also be worth briefly mentioning that other species have occasionally been reported as host plants (e.g. *Corylus avellana*, *Carpinus*, *Tilia*, *Ulmus*; e.g. Fajčík 2003, Robineau 2007).

 References

 [REF 1]

 [REF 2]

Are sufficient details of methods and materials provided to allow replication by others?

Yes

Is the rationale for creating the dataset(s) clearly described?

Yes

Are the datasets clearly presented in a useable and accessible format?

Yes

Are the protocols appropriate and is the work technically sound?

Yes

Reviewer Expertise:

Lepidoptera speciation, taxonomy, phylogeography

I confirm that I have read this submission and believe that I have an appropriate level of expertise to confirm that it is of an acceptable scientific standard.

## References

[1] Die Schmetterlinge Mittel- und Nordeuropas – Volumen 1. *https://www.insekten-egz.ch/literatur/die-schmetterlinge-mittel-und-nordeuropas-volumen-1/* .

[2] Guide to the moths of France. More than 1620 species described and illustrated (2nd edition). *https://www.researchgate.net/publication/320347214_Guide_des_papillons_nocturnes_de_France_Plus_de_1620_especes_decrites_et_illustrees_2eme_edition* .
